# Exogenous melatonin treatment reduces postharvest senescence and maintains the quality of papaya fruit during cold storage

**DOI:** 10.3389/fpls.2022.1039373

**Published:** 2022-12-06

**Authors:** Dengliang Wang, Mazhar Saeed Randhawa, Muhammad Azam, Hongru Liu, Shaghef Ejaz, Riadh Ilahy, Rashad Qadri, Muhammad Imran Khan, Muhammad Ali Umer, Muhammad Arslan Khan, Ke Wang

**Affiliations:** ^1^ 1Institute of Fruit Tree Research, Quzhou Academy of Agriculture and Forestry Science, Quzhou, China; ^2^ Pomology Laboratory, Institute of Horticultural Sciences, University of Agriculture, Faisalabad, Pakistan; ^3^ Institute of Crop Breeding & Cultivation Research, Shanghai Academy of Agricultural Sciences, Shanghai, China; ^4^ Department of Horticulture, Bahauddin Zakariya University, Multan, Pakistan; ^5^ Laboratory of Horticulture, National Agricultural Research Institute of Tunisia (INRAT), University of Carthage, Ariana, Tunisia; ^6^ Institute of Soil and Environmental Sciences, University of Agriculture, Faisalabad, Pakistan; ^7^ Key Laboratory of Jianghuai Agricultural Product Fine Processing and Resource Utilization, Ministry of Agriculture and Rural Affairs/Anhui Engineering Laboratory for Agro-products Processing, Anhui Agricultural University, Hefei, China

**Keywords:** Papaya, biochemical assays, antioxidant capacity, ROS, sensory attributes, postharvest storage, melatonin

## Abstract

**Introduction:**

Exogenous melatonin (EMT) application has been used to reduce postharvest senescence and improve the quality and antioxidant enzyme activities of papaya fruits during cold storage.

**Methods:**

The effects of exogenous melatonin application (1. 5 mM) were investigated on papaya fruits during cold storage (10°C ± 2°C) for 28 days in the present study.

**Results and discussion:**

The EMT treatment delayed postharvest senescence significantly with lower maturing status compared with untreated papaya fruits (control). In addition, EMT treatment maintained substantially higher titratable acidity values and ascorbic acid content but significantly lower soluble solids content and lower weight loss compared with the untreated fruits. Concerning the antioxidant capacity, the EMT-treated papaya fruit exhibited markedly higher total phenolic content and, consequently, higher DPPH-radical scavenging activity than the control group. The EMT treatment not only kept a higher enzyme activity of superoxide dismutase, peroxidase, and catalase but also significantly inhibited the accumulation of hydrogen peroxide and malondialdehyde, along with satisfying sensory attributes.

**Conclusion:**

The findings of this study indicated that EMT application could be commercially used as an eco-friendly strategy to reduce postharvest senescence and maintain the fresh-like quality traits of papaya fruit during cold storage.

## Introduction

Papaya (*Carica papaya* L.) is an economically important fruit that is mostly grown in tropical and subtropical zones of many countries (Wu et al., 2019). It is considered a rich source of bioactive compounds, phytochemicals, and minerals (Oloyede, 2005). Papaya is a climacteric fruit and ripens rapidly after postharvest. Several physical and biochemical changes cause pulp softening, skin discoloration, and fungal infection, significantly limiting papaya’s postharvest life (Escamilla-García et al., 2018; de Vasconcellos Santos Batista et al., 2020). Additionally, papaya fruits are prone to chilling injury during cold storage and fast quality deterioration during transportation due to the higher vulnerability of their delicate skin to mechanical damage (Nunes et al., 2006; Zerpa-Catanho et al., 2017). Papaya incurs up to 25% postharvest losses from the field to the retail level, where fungal diseases cause the majority (93%) of these losses. Similarly, 5% to 40% losses may occur during air and sea transit, depending on papaya handling and packaging methods (Nunes et al., 2006; Gajanana et al., 2010). Generally, ripe papaya fruits maintain fresh-like quality for 1 week at 25°C and for 14 days at 12°C (Wu et al., 2019). Most of the approaches adapted to control the postharvest deterioration of papayas either have high economic costs or require a high input of synthetic chemicals which posed a high risk to the environment and human health. Therefore, there is a need for an efficient and eco-friendly strategy to prolong the shelf life of papaya.

Recently, several methods, such as the application of putrescine (Hanif et al., 2020b), ozone (Ong et al., 2013a), bioactive extracts (Albertini et al., 2016) alone or combined with hot water treatment (Vilaplana et al., 2020), gamma and UV-C irradiation (Zhao et al., 1996; Cia et al., 2007), and edible coatings (Maqbool et al., 2011; Hamzah et al., 2013; Dotto et al., 2015; Dos Passos et al., 2020), have been extensively employed to enhance the postharvest storage quality of papaya fruits. Ong et al. (2013b) reported variations in physicochemical traits and antioxidant concentration during the postharvest ripening of papaya cv. Frangi. Moreover, the quality of fresh-cut products was kept by using postharvest green and innovative chemical treatments, which substantially inhibited microbial infestation (Ali et al., 2018). An increasing trend has recently been noticed which is using melatonin treatment to delay ripening and postharvest decay and to increase resistance against oxidative stress in stored fruits (Huang and Vanyo, 2020; Li et al., 2017). Fan et al. (2022) reported that melatonin application has safe commercial use in addition to enhancing storage potential and maintaining postharvest quality traits of various fruits.

Melatonin (N-acetyl-5-methoxytryptamine) has been implicated in abiotic and biotic stress tolerance in plants. Melatonin is an important pleiotropic molecule with multiple physiological and cellular actions in plants and is considered an efficient antioxidant and nutritional supplement for humans (Galano et al., 2011; Reiter et al., 2015; Sun et al., 2015; Liu et al., 2018; Yan et al., 2020). Wang et al. (2020) reported that melatonin is a ubiquitous signaling molecule involved in different plant growth and development processes, leading to improved crop yield and fruit storage life. Recently, the impact of exogenous melatonin (EMT) application to delay postharvest senescence and its effect on the biochemical and antioxidative defense system of fruits have gained extensive consideration. EMT application has been used to delay ripening and senescence, maintain quality, and increase postharvest life during storage in various fresh products such as tomato, peach, and strawberry fruits (Gao et al., 2016; Ma et al., 2016; Aghdam and Fard, 2017). EMT treatment also increased the endogenous melatonin level and improved the nutraceutical properties of various fruits including sweet cherries, bananas, and litchis (Hu et al., 2017; Wang et al., 2019; Wu et al., 2021). Melatonin treatments decreased fruit senescence by trapping reactive oxygen species (ROS) and malondialdehyde (MDA) content while improving the antioxidant activities and phenolic concentration (Li et al., 2017; Wang et al., 2019). EMT application significantly decreased the content of MDA and hydrogen peroxide (H_2_O_2_) and maintained higher superoxide dismutase (SOD), peroxidase (POD), catalase (CAT), and glutathione reductase (GR) enzyme activities than control and retained the integrity of the membrane wall in sweet cherry fruits (Sharafi et al., 2021). Wang et al. (2021) found that EMT application also delayed low-temperature injury, lowered peel browning, and increased the antioxidant defense system which contributed to lower H_2_O_2_ and O_2_^−^ production rates in banana fruit. Based on the abovementioned advantages, EMT treatment might be safe and eco-friendly and a better alternative to various chemical treatments in maintaining fresh-like fruit quality during storage. However, such studies on papaya fruit during cold storage are very limited and not complete.

Therefore, the aim of this research was to investigate the effect of EMT treatment in reducing postharvest senescence and maintaining the fresh-like quality of papaya fruit during cold storage (10°C for 28 days).

## Materials and methods

### Fruit materials and treatment

Papaya (*C. papaya* L. Cv. Red Lady) fruits were harvested at two physiological stages of maturation (with green and a trace of yellow) from a local orchard located at Faisalabad, Punjab, Pakistan, and kept under shade. Healthy and uniformly sized (450– 550 g) papaya fruits were selected and immediately delivered to the laboratory. The papaya fruits were washed with water to remove dirt before soaking in a disinfectant [sodium hypochlorite (0.1%, v/v)] solution for 2 min. Then, the fruits were rinsed and air-dried. For melatonin treatment, the fruits were divided into two groups. The first group of papaya fruits (control) was dipped only in distilled water, whereas the second group was treated with 1. 5 mM of melatonin for 10 min. A preliminary experiment on papaya fruits was conducted with four melatonin concentrations of 0.5, 1, 1.5, and 2 mM. Finally, 1. 5 mM of melatonin concentration was screened out for further experiments. The fruits were air-dried for 30 min and then stored at 10° C ± 2°C and 90%–95% relative humidity (RH) for 28 days after the treatments. Papaya fruits were sampled at 7-day intervals. Each treatment was comprised of three biological replications, each consisting of 20 fruits. The samples were frozen with liquid nitrogen and kept at −40°C for further analysis. The data were recorded at each sampling point through the storage period.

### Determination of decay incidence and weight loss

Papaya fruits with visible symptoms of rot and fungal or bacterial infections were considered as decay. In each treatment, the decay rate was measured by dividing the number of decaying fruits in relation to the total quantity of fruits. The results were expressed in percentage of decayed fruit. Regarding weight loss, three papaya fruits were marked and weighed at each interval using a digital electronic balance (EK-600H, Japan). The outcomes were presented as a percentage of weight reduction with respect to the initial weight.

### Determination of soluble solids content, titratable acidity, ripening index, and ascorbic acid content

The juice of the papaya fruits was prepared using a juicer machine (DN-DOB, DEURON, Japan) for chemical analysis. To determine the soluble solids content (SSC), a drop of juice was placed on the cleaned prism plate of a digital refractometer (RX 5000, Atago, Japan). Prior to taking reading, distilled water was used to calibrate the refractometer at room temperature (27°C). After measurements, distilled water was used to clean up the prism of the refractometer for further analysis and SSC was expressed as °Brix.

The measurement of titratable acidity (TA) was obtained according to the procedure of Hortwitz (1960). For TA measurement, 10 ml of fresh juice and 50 ml of distilled water were added to a 100-ml conical flask. TA was measured by the titration method using 0. 1 N of NaOH in the presence of phenolphthalein (one to two drops) as a pH indicator. Titration was continued until the achievement of the endpoint of pink color and the data were expressed as percent TA. The ripening index was determined by taking the ratio between SSC and TA (SSC: TA ratio).

The content of ascorbic acid (AsA) in papaya fruits was measured according to the method of Ruck (1961) by using 2, 6-dichlorophenolindophenol as a dye. For this, the samples were prepared by taking 5 ml of papaya juice extracted from three fruits per replication. Furthermore, 90 ml of 0.4% oxalic acid solution was added to the juice in a volumetric flask (100 ml). After filtration, an aliquot of 5 ml was titrated against the dye until a pink color appeared and remained at least for a period of 15 s. The results were expressed as mg of ascorbic acid per 100 ml of fruit juice.

### Determination of total sugar, reducing sugar, and non-reducing sugar (%)

Total, reducing, and non-reducing sugars of papaya fruits were evaluated using the standard method of Hortwitz (1960) by using a specified titration procedure.

### Measurement of total phenolic content and DPPH-radical scavenging activity

The total phenolic content in papaya fruit was estimated using the Folin–Ciocalteu reagent as suggested by Razzaq et al. (2013) with slight modification. The supernatant was extracted, vortexed, incubated, and then centrifuged at 13,000 × *g*. Finally, the absorbance was measured by using a spectrophotometer at 765 and 517 nm. The results were reported as mg of gallic acid equivalent (GAE)/100 g FW.

DPPH-radical scavenging activity (DPPH-RSA) was determined using the free radical 2,2-diphenyl-1-picrylhydrazyl as reported by Brand-Williams et al. (1995), and the results were expressed in percentage.

### Measurement of malondialdehyde and hydrogen peroxide contents

The determination of MDA content was conducted by using the thiobarbituric acid (TBA) assay (Hodges et al., 1999) with slight changes. Two grams of papaya pulp was mixed with 5 ml of 30 mM trichloroacetic acid and centrifuged (10,000 × *g* for 10 min at 4°C). The supernatant (1 ml) was mixed with 3 ml of 0.67% TBA and heated for 20 min. After cooling at room temperature, the absorbance was recorded at 450, 532, and 600 nm. MDA values were expressed as nmol g^−1^ FW. The measurement of H_2_O_2_ content was performed using the method of Ferguson et al. (1983) with minor modifications. Shortly, papaya pulp (1 g) was weighed, and 5 ml of precooled acetone was added to the sample, followed by homogenizing the sample and centrifuging (10,000 × *g*) for 10 min at 4°C. Afterward, 0.5 ml of the supernatant was mixed with precooled acetone (0. 5 ml), TiCl_4_–HCl (0. 1 ml, 10%), and concentrated ammonia (0. 2 ml). Then, the mixture was centrifuged (10,000 × *g* at 4°C for 15 min), the precipitate was then mixed with H_2_SO_4_ (3 ml, 2 mol L^−1^), and the absorbance of the solution was measured at 412 nm. H_2_O_2_ production rate was expressed as mmol kg^−1^.

### Determination of antioxidant enzyme activities

For the determination of antioxidant enzyme activities, the papaya pulp sample (1 g) was homogenized with phosphate buffer (pH 7.2), and then the homogenate was centrifuged at 10,000 × *g* for 10 min at 4°C. The supernatant was collected for further assessment of enzymatic activities.

The SOD activity of papaya was determined according to the method of Stajner and Popovic (2009) with slight modifications. Shortly, the reaction mixture consisted of 500 μl of phosphate buffer (50 mM, pH 5), 200 μl of methionine (22 μM), 100 μl of NBT (20 μM), 200 μl of Triton X (0. 1 μM), and 100 μl of riboflavin (0. 6 μM), along with 800 μl of distilled water and 100 μl of enzyme extract. Then, the mixture was incubated in a box under luminous lamps for 15 min. The changes in absorbance were monitored and recorded at 560 nm, and SOD activity was calculated as U mg^−1^ protein. The POD activity of papaya fruit extract was determined according to Liu et al. (2014) with minor changes. Briefly, the reaction mixture, containing 0. 5 ml of crude extract and 2 ml of guaiacol substrate, was incubated at 30°C for 5 min, and 1 ml of H_2_O_2_ (24 mM) was added to it. The change in absorbance at 460 nm was monitored, and POD activity was expressed as U mg^−1^ protein. Catalase activity was measured as outlined by Lafuente et al. (2004). The reaction mixture, comprised of enzyme extract (100 μl), 50 mM of sodium phosphate buffer (pH 7.0), and 130 mM of H_2_O_2_, was incubated at 37°C for 1 min. Furthermore, ammonium molybdate (32. 4 mM) was added to the mixture and shaken for 1 min. The absorbance fluctuation of 0. 01 units per minute was considered as one catalase activity unit (U mg^−1^ protein).

### Sensory evaluation

The changes in the appearance, staste, aroma, and sweetness of papaya fruits during the storage period were evaluated by using a 9-point hedonic scoring scale (Lawless and Heymann, 2010). Papaya fruits were peeled off, sliced, and presented to the trained panel on randomly arranged white plates for the scoring. The hedonic score points varied from 1 = dislike extremely and 5 = neither like/nor dislike to 9 = like extremely. However, to indicate the acceptability of the fruit, a score of ≥ 6 “I like it slightly” was considered.

### Statistics

The experiment was conducted according to a completely randomized block design with the factorial arrangement. The data were subjected to the analysis of variance (ANOVA) procedure by using Statistix 8.1 (USA) statistical software, and significant differences among treatment means were calculated using the least significance difference test (*P* ≤ 0.05).

## Results

### Effect of EMT treatment on decay incidence and weight loss rate

**Figure 1:**
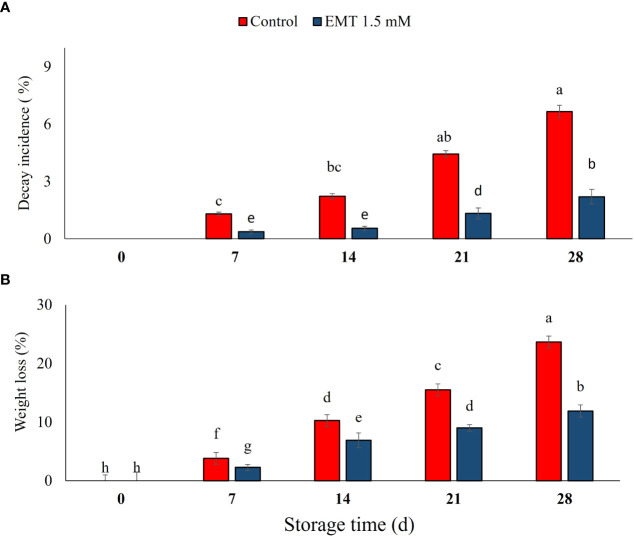
Effect of exogenous melatonin (EMT) treatment on fruit decay **(A)** and weight loss **(B)** of papaya fruits during cold storage. Data collected from the mean of three replicates, and vertical bars indicate the standard error of the means. Mean values with different letters show significant differences, and those with similar letters show no statistical difference according to the least significant difference test (*P* < 0.05).

In the present study, the decay incidence gradually increased across the two treatments in papaya fruits during storage. However, a higher decay incidence was recorded in the control than in EMT-treated papaya fruits (*P* < 0.05). Moreover, EMT treatment substantially reduced fruit decay, and the incidence was 66% lower than that of untreated fruits on 28 days of cold storage (**Figure 1A**). Irrespective of the treatments, there was an increased weight loss in papaya fruits during postharvest storage (**Figure 1B**). However, the EMT-treated fruits exhibited significantly reduced weight loss (49% less) compared with the control on 28 days of storage.

### Effect of EMT treatment on soluble solids content, titratable acidity, ripening index, and ascorbic acid content

**Figure 2:**
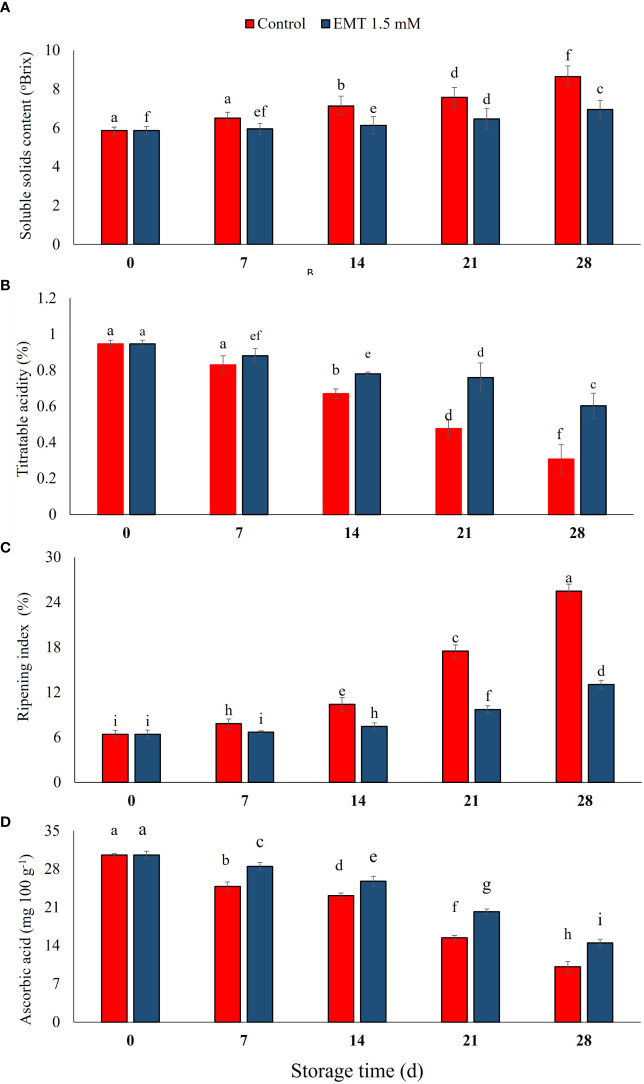
Effect of EMT treatment on soluble solids content **(A)**, titratable acidity **(B)**, ripening index **(C)**, and ascorbic acid **(D)** of papaya fruits during cold storage. Data collected from the mean of three replicates, and vertical bars indicate the standard error of the means. Mean values with different letters show significant differences, and those with the same letters show no statistical difference according to the least significant difference test (*P* < 0.05).

The SSC content increased regardless of treatments throughout the storage duration (**Figure 2A**). However, the EMT-treated papaya fruits showed a substantially (*P* < 0.05) limited increase with respect to the control fruits throughout the storage. The EMT-treated fruits had 20% lower SSC content than the control group on 28 days of storage. Overall, melatonin treatment inhibited SSC accumulation in papaya fruits (**Figure 2A**). Moreover, EMT treatment significantly decelerated the decrease in TA during the whole storage period (*P* < 0.05). The EMT-treated papaya fruits exhibited significantly higher (96% higher) TA values than the control group on 28 days of storage (**Figure 2B**).

Following EMT application, the ripening index was significantly (*P* < 0.05) reduced in treated papaya fruits during the storage duration (**Figure 2C**). The EMT-treated fruits showed a lower (14% to 48%) ripening index from 7 to 28 days of storage as compared with the untreated fruits, respectively. AsA content exhibited a decreasing trend regardless of the applied treatments (**Figure 2D**). However, EMT treatment maintained a considerably (*P* < 0.05) higher (14% to 43%) concentration of ascorbic acid than the control group between 7 and 28 days of storage.

### Effect of EMT treatment on total sugar, reducing sugar, and non-reducing sugar

**Figure 3:**
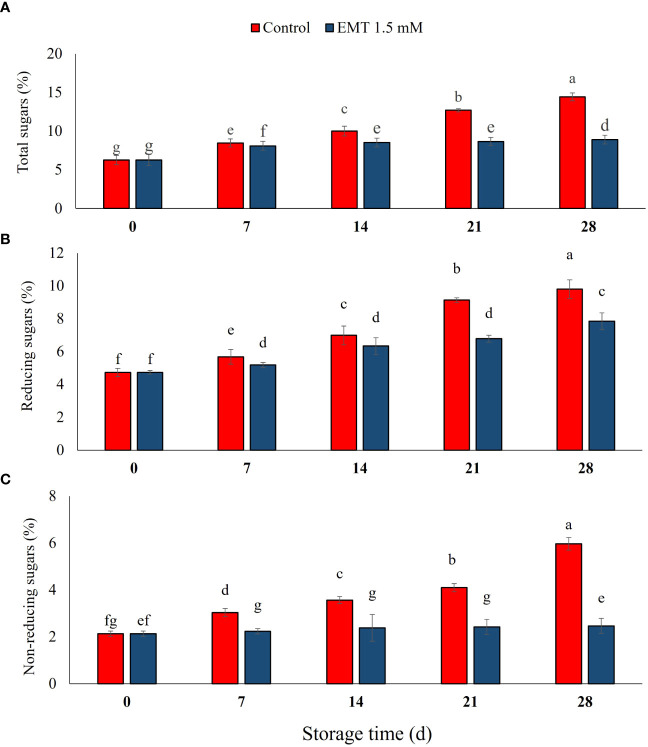
Effect of EMT treatment on total sugar **(A)**, reducing sugar **(B)**, and non-reducing sugar **(C)** of papaya fruits during cold storage. Data collected from the mean of three replicates, and vertical bars indicate the standard error of the means. Mean values with different letters show significant differences, and those with the same letters show no statistical difference according to the least significant difference test (*P* < 0.05).

Total sugar, reducing sugar, and non-reducing sugar contents changed gradually in papaya fruits throughout the storage period (*P* < 0.05; **Figures 3A–C**). All the three kinds of sugar content were increased in the control during the storage, while the EMT-treated fruits were not changed so obviously except for 7 and 28 days as compared with the 0 days. The EMT-treated fruits had considerably less total sugar (38%), reducing sugar (19%), and non-reducing sugar (59%) as compared with the control fruits on 28 days of storage (**Figures 3A–C**).

### Effect of EMT treatment on malondialdehyde and hydrogen peroxide content

**Figure 4:**
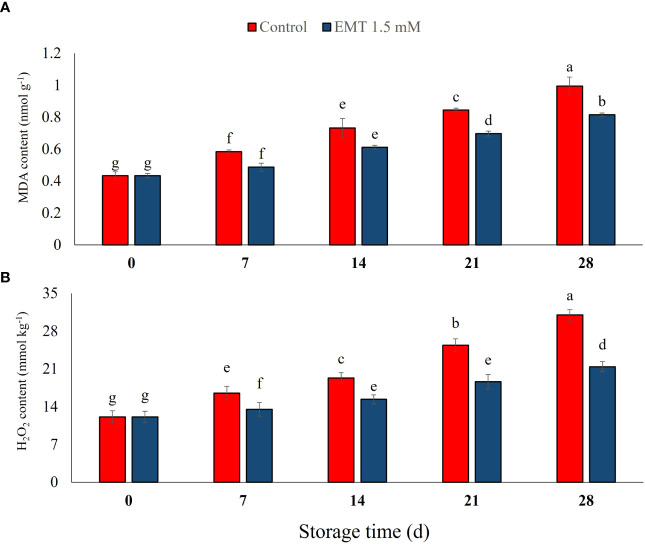
Effect of EMT treatment on MDA **(A)** and H_2_O_2_ content **(B)** of papaya fruits during cold storage. Data collected from the mean of three replicates, and vertical bars indicate the standard error of the means. Mean values with different letters show significant differences, and those with the same letters show no statistical difference according to the least significant difference test (*P* < 0.05).

In this study, MDA content increased significantly during the storage, while the EMT treatment significantly inhibited the accumulation of MDA compared with the control from 14 to 28 days (*P* < 0.05; **Figure 4A**). In addition, the EMT-treated papaya fruits showed 18% lower MDA content with respect to the untreated fruits on 28 days of cold storage. As shown in **Figure 4B**, the H_2_O_2_ contents were increased in both EMT-treated papaya and control fruits throughout the cold storage period. However, the EMT-treated papaya fruits delayed the increase of H_2_O_2_ contents and exhibited 30% lower H_2_O_2_ contents than the control group on 28 days of storage (*P* < 0.05; **Figure 4B**).

### Effect of EMT application on total phenolic content and DPPH-RSA

**Figure 5:**
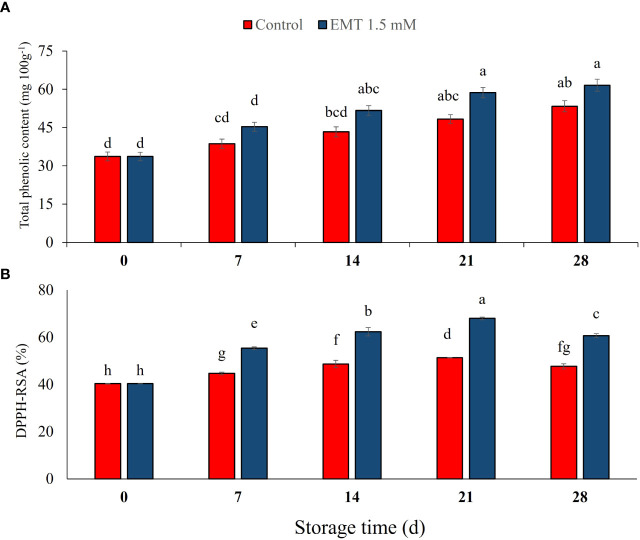
Effect of EMT treatment on total phenolic content **(A)** and DPPH-RSA activity **(B)** of papaya fruits during cold storage. Data collected from the mean of three replicates, and vertical bars indicate the standard error of the means. Mean values with different letters show significant differences, and those with the same letters show no statistical difference according to the least significant difference test (*P* < 0.05).

Total phenolic contents (TPCs) were moderately enhanced from 7 to 28 days in both EMT-treated and untreated fruits (*P* < 0.05). However, the EMT-treated papaya fruits displayed 15% higher TPC than the untreated fruits on 28 days of storage (**Figure 5A**). DPPH-RSA activity in the stored papaya fruits was significantly (*P* < 0.05) increased up to 21 days and then decreased up to 28 days of storage. However, DPPH-RSA activity was significantly higher in the EMT-treated fruits and peaked on 21 days of storage (33% higher) as compared with that of the untreated fruits (**Figure 5B**). DPPH-RSA in the EMT-treated papaya fruits remained 27% higher than in the untreated fruits on 28 days of storage.

### Effect of EMT treatment on antioxidant enzymes

**Figure 6:**
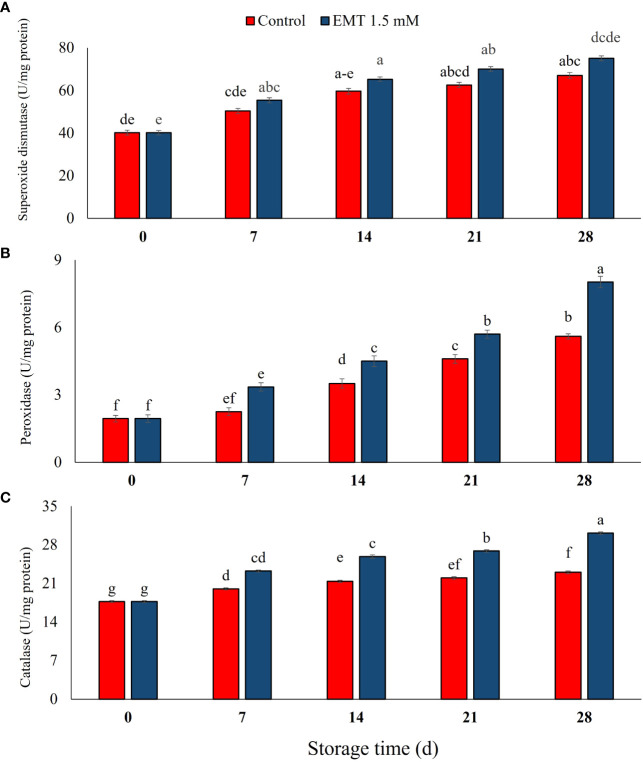
Effect of EMT treatment on superoxide dismutase (U mg^−1^ protein) **(A)**, peroxidase (U mg^−1^ protein) **(B)**, and catalase (U mg^−1^ protein) **(C)** of papaya fruits during cold storage. Data collected from the mean of three replicates, and vertical bars indicate the standard error of the means. Mean values with different letters show significant differences, and those with the same letters show no statistical difference according to the least significant difference test (*P* < 0.05).

The activity of SOD substantially (*P* < 0.05) increased in the EMT-treated papaya fruits compared with the control group during the storage period (**Figure 6A**). EMT treatment enhanced SOD activity by almost 14% compared with the control papaya fruits on 28 days of storage. Peroxidase activity gradually increased in both EMT-treated and control fruits during the entire storage duration (**Figure 6B**). However, the EMT-treated fruits showed significantly higher (25%) levels of POD activity than the untreated fruits on 28 days of storage (*P* < 0.05). CAT activity significantly increased during the storage except for the interval between 7 and 14 days in the EMT-treated fruits, while the activity only increased slightly from 0 to 14 days in the control (**Figure 6C**). The EMT treatment significantly induced CAT activity from 14 to 28 days of storage and promoted CAT activity by 12% as compared with the control on 28 days of storage (*P* < 0.05; **Figure 6C**).

### Effect of EMT treatment on sensory attributes

**Figure 7:**
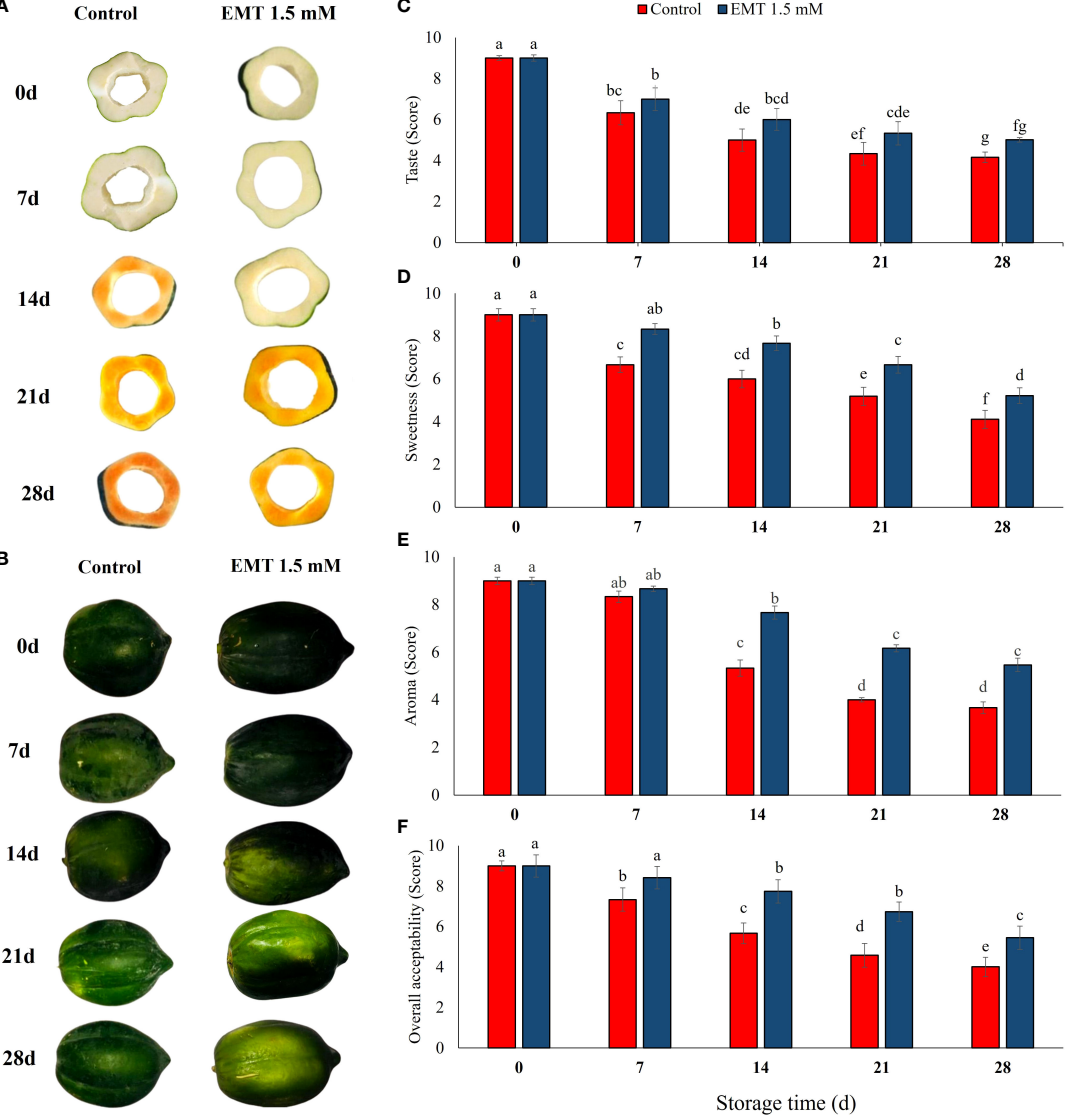
Effect of EMT treatment on visual quality **(A, B)**, taste **(C)**, sweetness **(D)**, aroma **(E)**, and overall acceptability **(F)** of papaya fruits during cold storage. Data collected from the mean of three replicates, and vertical bars indicate the standard error of the means. Mean values with different letters show significant differences, and those with the same letters show no statistical difference according to the least significant difference test (P < 0.05).

The EMT-treated fruits had significantly improved sensory attributes such as taste, sweetness, aroma, and overall acceptability as compared with the untreated papaya fruits on 28 days of storage (**Figures 7A–F**). While most of the sensory attributes gradually decreased in both EMT-treated and control papaya fruits, the EMT-treated papaya fruits maintained higher taste (38%), sweetness (28%), aroma (49%), and overall acceptability (36%) than the untreated fruits at the end of the storage period (*P* < 0.05; **Figures 7C–F**).

### Correlation analysis

**Figure 8:**
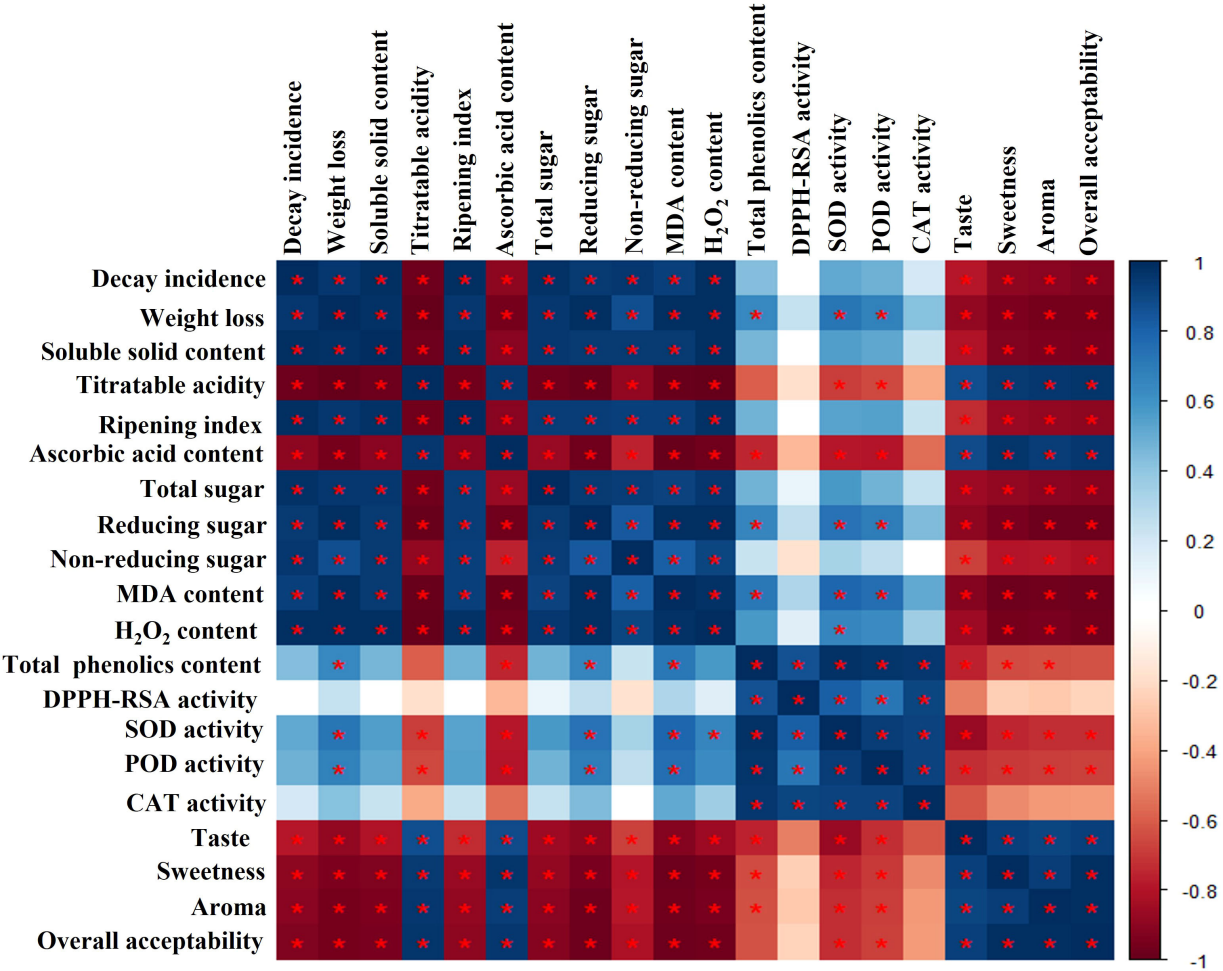
Correlation heat map analysis of each variable of EMT-treated papaya fruits during cold storage. The intensity of color represents the strength of the correlation: the blue color indicates a positive correlation, and the red color displays a negative correlation. The symbol * expresses significant correlations.

The correlation analysis of different parameters showed that decay incidence was positively correlated with weight loss, soluble solids contents, ripening index, total sugar, reducing sugar, non-reducing sugar, MDA, H_2_O_2_, SOD, POD, and CAT (*P* < 0.05; **Figure 8**). Moreover, decay incidence was significantly negatively correlated with titratable acidity, ascorbic acid, taste, sweetness, aroma, and overall acceptability **(** *P* < 0.05; **Figure 8**). Our results suggest that EMT treatment substantially reduced decay incidence and weight loss rate and maintained the higher antioxidant enzyme activities (SOD, POD, CAT) by inhibiting the rate of MDA and H_2_O_2_ contents. This indicated that EMT treatment maintained the sensory quality and prolonged the shelf life of papaya during cold storage.

## Discussion

Mostly, postharvest decay incidence and weight loss occur because of increased respiration rate and fungal pathogen infection in papaya fruits. Different fungicides have been used to manage and control fruit decay; however, consumers are increasingly concerned about the use of synthetic chemicals because of their negative effects (Hanif et al., 2020a). Consequently, melatonin, a ubiquitous molecule, has shown great potential for extending the postharvest life of fresh produces (Nawaz et al., 2016; Aghdam et al., 2019; Tiwari et al., 2020; Bose and Howlader, 2020). Fruit decay during storage is one of the major problems for the fresh food industry, which results in high losses and inferior fruit quality, hampering the fruits’ marketability. Higher respiration and ethylene production rapidly increase weight loss in fruits and vegetables during storage. Likewise, Ong et al. (2013b) reported a significant increase in weight loss during the postharvest ripening of papaya. In the present study, EMT treatment significantly decreased the senescence and weight loss rate in comparison to the control during the whole storage period. Similar findings have been reported, where EMT-treated strawberry fruits exhibited substantially lower senescence and weight loss rates during cold storage (Liu et al., 2018). In papaya fruits (Hanif et al., 2020b) and cherries (Correia et al., 2017), weight loss increases during storage due to higher respiration and evaporation rate and fast utilization of stored metabolites for cellular metabolic activities. Likewise, Gao et al. (2016) reported that exogenous EMT treatment markedly reduced peach quality deterioration and weight loss during storage. Recently, studies have demonstrated that EMT treatment significantly reduced weight loss, delayed senescence, and improved the resistance against abiotic stress in sweet cherry fruits (Wang et al., 2019).

In this study, melatonin treatment effectively maintained higher titratable acidity and ascorbic acid content during storage, whereas it significantly reduced the change in SSC, ripening index, and sugar contents in stored papaya fruits. Our results agreed with the earlier studies of Liu et al. (2018) who noticed that EMT treatment at 0. 1 mM substantially reduced SSC and higher acidity which contributed to the delay in the senescence of strawberry fruits under cold storage conditions. Similarly, Gao et al. (2016) determined that EMT treatment significantly maintained the levels of ascorbic acid content in peach fruits as compared with the control. Likewise, ozone treatments effectively reduce the microbial population and maintained the quality traits of papaya fruit during storage (Kying and Ali, 2016). Bal (2019) reported that exogenous EMT (1 mM) treatment on plum fruits caused lower SSC and higher titratable acidity values which contributed to the slowing down of senescence in the treated fruits. The sugar–acid ratio is considered an important indicator of fruit flavor and consumer acceptance and appreciation (Gao et al., 2016). Meanwhile, a low ripening index indicated the suitability of treated fruits for a prolonged storage period. In our study, the EMT-treated fruits led to reduced ripening index values and a slow ripening process which resulted in an extended storage life of the papaya fruit. This is in agreement with previous studies on papaya and plum fruits (Bal, 2019; Hanif et al., 2020b).

Starch breakdown increases the accumulation of simple sugars during the ripening of fruits (Hanif et al., 2020b). In this study, total, reducing, and non-reducing sugars exhibited a higher accumulation trend in the control than in the EMT-treated fruits. A lower accumulation of sugars is an important indicator of delayed postharvest ripening and senescence due to the inhibition of amylase and phosphorylase enzyme activity (Hanif et al., 2020a). In addition, sugars influenced fruit flavor and consumers’ acceptability for sweetness, and organic acids contributed to the increase of sugar levels as respiratory substrates (Hu et al., 2017).

Total antioxidants and TPC are important nutritional quality traits for stored fruits and vegetables. Our results showed that following EMT treatment, TPC content and DPPH scavenging activity gradually increased and remained high during the entire storage. Ghasemnezhad et al. (2010) reported that chitosan-coated peach fruits with higher TPC and DPPH scavenging activity showed higher anti-senescence properties than the control group during cold storage. The salicylic acid treatment increased the levels of TPC and DPPH scavenging activity in papaya fruits stored at 12°C for 28 days (Hanif et al., 2020b). The EMT treatment increased DPPH scavenging potential that ultimately stimulated non-enzymatic antioxidative defense levels. EMT application delayed senescence and led to a higher accumulation of TPC and flavonoid compounds that induced a higher antioxidant potential and inhibited the synthesis of H_2_O_2_ in stored strawberry (Liu et al., 2018), sweet cherry (Sharafi et al., 2021), and tomato fruits (Sharafi et al., 2019) under cold storage. Recently, various researchers have suggested that higher activities of phenylpropanoid pathway enzymes along with lower activities of polyphenoloxidase enzymes improve phytochemical accumulation and DPPH scavenging capacity in fresh produce (Gao et al., 2016; Aghdam et al., 2019).

Exogenous melatonin application plays an important role in enhancing the activity of ROS scavenging enzymes that combat various ROS, thereby maintaining the membrane’s integrity within fruit tissues (Zhao et al., 2013; Zhang et al., 2018). Antioxidant enzymes (SOD, POD, CAT, APX) regulate lipid peroxidation and accumulation of ROS (Xu et al., 2019). Enhanced antioxidant enzyme activities may reduce the peroxidation of lipids which consequently delays senescence in peach fruits (Flores et al., 2008). In the present study, EMT application promoted the activity of antioxidant enzymes in stored papaya fruits. Similar results have been observed by Gao et al. (2016), who reported that delayed fruit decay and higher antioxidant enzyme activities were related to the increased cell wall integrity and lower cell wall- degrading enzyme activities in EMT-treated peach fruits. Likewise, our findings are in line with previous studies whereby SOD, POD, and CAT activities increased in sweet cherries following EMT treatment (Wang et al., 2019). A similar result was also obtained by Arnao and Hernandez-Ruiz (2018), who reported that EMT treatment increased antioxidant enzyme activities and reduced the biosynthesis of ROS which inhibited ethylene biosynthesis and, consequently, halted the fruit ripening process. A similar observation has been reported in blueberry and pear (Zhai et al., 2018; Shang et al., 2021) and banana (Hu et al., 2017) fruits during storage. So, EMT treatment reduced oxidative stress which contributed to slowing down senescence and maintaining the fresh-like quality of papaya fruit for an extended storage duration.

The disintegration of cellular membranes is highly related to elevated electrolyte leakage and, consequently, rapid fruit senescence (Duan et al., 2011). Excessive accumulation of MDA levels is often related to the loss of cellular membrane integrity during fruit storage (Duan et al., 2011; Yang et al., 2014). The activities of antioxidant enzymes in plants are important in reducing the level of MDA, delaying senescence, and maintaining cellular redox status (Jimenez et al., 2002). In the present study, EMT treatment reduced H_2_O_2_ and MDA levels as compared with the untreated papaya fruits. Melatonin was considered an antioxidant compound which improved the storage quality and life of cherry fruits by limiting oxidative stress (Yu et al., 2013). In the same context, Shang et al. (2021) reported that EMT treatment significantly decreased H_2_O_2_ and MDA biosynthesis, the main oxidative stress markers, which delayed senescence in blueberry fruits during storage. Recently, Xu et al. (2019) reported multifaceted mechanisms, following the EMT treatment of fruits and vegetables, such as ROS neutralization, an increase in antioxidant enzyme activities and non-enzymatic antioxidant levels, and protein repairs. It has been reported that the loss of subcellular compartmentalization following cellular breakdown resulted in higher utilization of phenolics as substrates and led to the reduction of postharvest storage quality of various horticultural produces (Yang et al., 2014; Wang et al., 2019). Similarly, Fu et al. (2017) reported that EMT treatment of *Elymus nutans* improved the antioxidant enzyme activities, suppressed the MDA content, and exhibited higher tolerance to cold stress.

Sensory attributes are important quality parameters for consumers’ acceptance of fruits and vegetables (Radi et al., 2017). In our study, EMT application maintained higher sensory quality traits compared with the untreated papaya fruits. Generally, there is a loss of consumer acceptance for fresh produce following unsatisfactory sensory quality or a decline in phytochemical and nutritional quality, especially after storage. Liu et al. (2018) noticed lower SSC and higher titratable acidity which significantly delayed the postharvest senescence process and preserved the sensory attributes of EMT-treated strawberry fruits stored at 4°C. Similarly, Onik et al. (2021) noticed a better skin appearance of stored apples following EMT treatment. Our results are in agreement with the studies on peaches (Cao et al., 2018), pears (Zhai et al., 2018), and banana fruits (Hu et al., 2017), which noticed that EMT treatments significantly delayed decay incidence and senescence, improved nutritional profiles, and conserved the sensory attributes. Recently, Wang et al. (2019) reported that EMT treatment may lead to a higher accumulation of endogenous melatonin and phenolic compounds, an improvement in overall nutritional quality, and a decline in H_2_O_2_ and MDA concentrations in sweet cherry fruits. The application of EMT enhanced the shelf life of fresh produces and was generally recognized as safe for human health (Zhai et al., 2018; Ze et al., 2021). Therefore, EMT treatment might be an efficient tool for boosting ROS scavenging abilities and improving the quality attributes of papaya fruits during cold storage.

## Conclusion

In this study, the comprehensive beneficial effects of EMT treatment on papaya fruit quality were evaluated, including the assessment of the main physicochemical traits, bioactive compound content, antioxidant activity, and ROS scavenging enzyme activities during cold storage. EMT application effectively delayed postharvest senescence, reduced weight loss, and maintained higher TA, lower SSC, and ripening index compared with the control. Furthermore, EMT application stimulated the accumulation of phenolic content and higher DPPH-scavenging abilities in papaya fruits. In addition, EMT treatment markedly suppressed the levels of ROS and MDA by enhancing the antioxidant enzyme activities in papaya fruits during cold storage. Therefore, EMT application is a promising method for delaying senescence, maintaining quality attributes, and extending the postharvest life of papaya during cold storage.

## Data availability statement

The original contributions presented in the study are included in the article/supplementary material. Further inquiries can be directed to the corresponding authors.

## Author contributions

DW, MSR, MA, KW: Data curation, Formal analysis, Investigation, Methodology, Writing–original draft. MA, SE, HL: Conceptualization, Funding acquisition, Methodology, Supervision, Writing – review and editing. RI, SE, RQ, MAU: Formal analysis, Investigation, Methodology, Writing – original draft. MIK, RQ, MAK: Methodology, Writing – review and editing. All authors contributed to the article and approved the submitted version.
